# Effects of Four Organic Carbon Sources on the Growth and Astaxanthin Accumulation of *Haematococcus lacustris*

**DOI:** 10.3390/life14010029

**Published:** 2023-12-24

**Authors:** Huijeong Byeon, Yunji An, Taesoo Kim, Vijay Rayamajhi, Jihyun Lee, HyunWoung Shin, SangMok Jung

**Affiliations:** 1 Department of Biology, Soonchunhyang University, Asan 31538, Chungcheongnam-do, Republic of Korea; emily2637@naver.com (H.B.);; 2 Korea Fisheries Resources Agency East Sea Branch, Samho-ro, Buk-gu, Pohang 37601, Gyungsangbuk-do, Republic of Korea; 3 AlgaeBio, Inc., Asan 31459, Chungcheongnam-do, Republic of Korea; 4 Research Institute for Basic Science, Soonchunhyang University, Asan 31538, Chungcheongnam-do, Republic of Korea

**Keywords:** *Haematococcus lacustris*, astaxanthin, sodium acetate, glycerol, microalgae

## Abstract

The microalga *Haematococcus lacustris* has a complex life cycle and a slow growth rate, hampering its mass cultivation. Culture of microalgae with organic carbon sources can increase the growth rate. Few studies have evaluated the effects of organic carbon sources on *H*. *lacustris*. We compared the vegetative and inductive stages of *H*. *lacustris* under autotrophic and mixotrophic conditions using four organic carbon sources: sodium acetate, glycerol, sodium gluconate, and ribose, each at various concentrations (0.325, 0.65, 1.3, and 2.6 g/L). The cell density was increased by 1.3 g/L of glycerol in the vegetative stage. The rapid transition to the inductive stage under nitrogen-depletion conditions caused by 1.3 or 2.6 g/L sodium acetate promoted the accumulation of astaxanthin. The production of astaxanthin by *H*. *lacustris* in mass culture using organic carbon sources could increase profitability.

## 1. Introduction

Carotenoids are organic pigments synthesized by plants, algae, bacteria, and fungi and have more than 700 structural varieties [1]. The ketocarotenoid astaxanthin (3,3′-dihydroxy-β, β’-carotene-4,4′-dione) is a red pigment with antioxidant activity as a result of its hydroxyl and keto groups [2]. Astaxanthin is used as a raw material for pharmaceuticals and functional foods because it suppresses inflammation, is nontoxic, and prevents diabetes and cancer [2,3]. By 2027, the market value of astaxanthin is expected to increase to USD 3.4 billion [4]. Astaxanthin may be synthesized chemically or extracted from natural sources. The synthetic version contains isomers that are different from those in natural products; these have low biological stability [5,6]. Synthetic astaxanthin is not approved as a food supplement [7] and is less effective than astaxanthin from an algal source [8]. Natural astaxanthin has approximately 20- to 50-fold greater antioxidant activity than the synthetic version [4]. It is biosynthesized by several bacteria, fungi, and plants; the edible product is mainly produced by the microalgae *Haematococcus lacustris*, *Chromochloris zofingiensis*, *Halochlorella rubescens*, *Ettlia carotinosa*, and *Rhexinema sarcinoideum* [8]. The green alga *Haematococcus lacustris* is an important source of astaxanthin, which can make up to 4% of the algae components under natural light and 8% under artificial light [7]. At 80–99% of total carotenoids, astaxanthin exists in a highly pure form in *Haematococcus* [9]. Astaxanthin has a polyene system and can exist in 9-*cis*, 13-*cis*, 15-*cis*, and all-*trans* geometric isomeric forms [10]. The *trans*-isomeric form is more stable than the *cis*-isomeric form [4]. The 3S, 3′S stereoisomer of astaxanthin is suitable for humans. Chemical synthesis yields a stereoisomeric ratio of 1(3S, 3′S):2(3R, 3′S):1(3R, 3′R). The 3S, 3′S form is produced by *H*. *lacustris* [11]. The life cycle of *Haematococcus* is divided into four stages: green vegetative cells, macrozooids, microzooids, and haematocysts; the latter accumulate astaxanthin [8,9,10]. At the green vegetative stage, motile and palmelloid cells can proliferate into 2–32 daughter cells via mitosis [12,13,14]. However, growth is reduced, and cells increase in size under stress conditions such as adverse temperatures, pH values, salinities, light intensities, or nutrient deprivation. Lipid droplets are formed in the cytoplasm, accumulating a large amount of astaxanthin, giving them a characteristic bright red color [10]. *Haematococcus* has a slower growth rate compared to most other microalgae; the doubling time is 85 to 192 h, depending on the medium and light intensity [15].

Organic carbon sources in mixotrophic culture promote cell division, haematocyst production, and carotenoid accumulation [16]. Inorganic carbon sources directly affect microalgal growth as a result of the high concentration of CO_2_ gas and pH control [17]. Astaxanthin production can be promoted by increasing the concentration of CO_2_; ≤5% CO_2_ favored the growth and astaxanthin accumulation of *Haematococcus*, whereas higher concentrations of CO_2_ suppressed growth [18,19,20,21]. Astaxanthin assimilation can be enhanced by adding carbon sources such as acetate, ribose, and mannose to mixotrophic cultures. However, few studies have evaluated the effects of external carbon sources on the growth rate and astaxanthin accumulation of *H*. *lacustris*. The benefits of CO_2_ injection are debated. Efforts to improve the growth rate by adding external carbon sources are environmentally and economically necessary. We evaluated the effects of four external carbon sources on the biomass and astaxanthin productivity of *H*. *lacustris* in the presence of natural air. The results may provide guidance for the large-scale cultivation of *H. lacustris*. Therefore, the most important considerations were sustainability and economic viability. We used sodium acetate, sodium gluconate, and glycerol, which are inexpensive, as external carbon sources. In addition, we examined ribose, despite its high cost, to determine whether its application is profitable. We evaluated the effects of these four sources on the growth and astaxanthin accumulation of *H*. *lacustris*.

## 2. Materials and Methods

### 2.1. Strain and Culture Conditions

*H. lacustris* (LIMS-PS-1354) was obtained from the Library of Marine Samples at KIOST, Geoje, South Korea. *H. lacustris* was cultured at 21 ± 1 °C with a 12:12 h light:dark cycle and a photon flux density of 37 μmol m^−2^ s^−1^ in Jaworski’s medium (JM). To prepare JM, six stock solutions were generated by mixing 10.0 g MgSO_4_·7H_2_O, 4.0 g Ca(NO_3_)_2_·4H_2_O, 2.48 g KH_2_PO_4_, 3.18 g NaHCO_3_, 7.2 g Na_2_HPO_4_·12H_2_O, and 16 g NaNO_3_ in 200 mL deionized water. Similarly, a stock solution was prepared by mixing 0.45 g EDTAFeNa and 0.45 g EDTANa_2_ in 200 mL deionized water. Another was prepared by mixing 0.496 g H_3_BO_3_, 0.278 g MnCl_2_·4H_2_O, and 0.20 g (NH_4_)_6_Mo_7_O_24_·4H_2_O in 200 mL deionized water. The last stock solution was prepared by mixing 0.008 g cyanocobalamin, 0.008 g thiamine HCl, and 0.008 g biotin in 200 mL deionized water. Finally, 1 mL of each stock solution was made up to 1 L with deionized water, creating the JM (pH 7) [22]. To compare effects on growth, 50 mL of cell suspension was inoculated into 150 mL of the JM in a 250 mL Erlenmeyer flask.

To evaluate astaxanthin accumulation, cell suspension (6.82 × 10^4^ mL^−1^) was centrifuged at 3000 rpm for 15 min and washed twice with distilled water. Next, 20 mL of the culture was inoculated into a 250 mL Erlenmeyer flask containing nitrogen-depleted JM medium. Subsequently, 20 mL of culture was diluted by adding water up to 180 mL. For nitrogen starvation, the nitrogen sources Ca(NO_3_)_2_·4H_2_O and NaNO_3_ were not added during the preparation of the JM.

### 2.2. Organic Carbon Sources

Sodium acetate (≥99%, JT Baker, Phillipsburg, NJ, USA), glycerol (≥99%, Sigma-Aldrich, Burlington, NJ, USA), sodium gluconate (≥99%, Sigma-Aldrich, Burlington, NJ, USA), and D-ribose (≥99%, ACROS Organics, Geel, Belgium) were used as organic carbon sources. Each of these was tested at concentrations of 0.325, 0.65, 1.3, and 2.6 g/L [23,24].

### 2.3. Analysis of Cell Growth

Cell concentrations were measured at 3-day intervals, and growth curves were plotted. Dry weight (DW) was measured after 14 days of culture. Cell density was measured using a haemocytometer (Neubauer-Improved with Dark-Line, Marienfeld, Germany) under a light microscope (×20) (BX53, Olympus, Japan). Absorbance at a wavelength of 680 nm was measured using a UV-vis spectrophotometer (UV-1601, Simadzu, Kyoto, Japan). To determine DW, 3 mL aliquots of culture were centrifuged for 5 min at 2000 rpm. The cell pellets were washed twice with distilled water, dried in a pre-weighed aluminum dish at 105 °C for 24 h until a constant weight was reached, cooled to room temperature in a desiccator, and weighed. Weights were converted into grams per liter.

### 2.4. Analysis of Total Carotenoid Content

To evaluate astaxanthin accumulation, total carotenoid content was measured after 9 days of culture under nitrogen-depleted conditions. For this purpose, 10 mL of *H*. *lacustris* suspension was transferred to a 15 mL conical tube and centrifuged at 4500 rpm for 15 min. After removing the supernatant, 10 mL of dimethyl sulfoxide (DMSO) was added, followed by boiling at 55 °C for 10 min and vortexing for 30 s to disrupt cells. Samples were centrifuged, and the absorbances (A) at wavelengths of 665, 649, and 480 nm of the supernatants were measured using a spectrophotometer. Carotenoid content was calculated using the formula of Wellburn (1994) [25]:

C*_a_* (µg mL^−1^) = 12.19 A_665_ − 3.45 A_649_

C*_b_* (µg mL^−1^) = 21.99 A_649_ − 5.32 A_665_

Total carotenoids (µg mL^−1^) = (1000 A_480_ − 2.14 C*_a_* [µg mL^−1^] − 70.16 C*_b_* [µg mL^−1^]) ÷ 220

where C*_a_* is chlorophyll *a*; C*_b_* is chlorophyll *b*; and A_665_, A_649_, and A_480_ are the absorbances at 665, 649, and 480 nm, respectively.

### 2.5. Analysis of Astaxanthin Content

To evaluate the astaxanthin content, 10 mL samples were transferred to conical tubes and centrifuged at 1700 g for 10 min. The pellets were treated with 5% (*w*/*v*) KOH diluted with 30% (*v*/*v*) methanol. Chlorophyll was degraded at 70 °C for 10 min, and samples were centrifuged at 3500 rpm for 10 min. The supernatants were removed, and 100 µL of glacial acetic acid was added. Subsequently, 5 mL of DMSO was added, and the solutions were boiled at 70 °C for 15 min. After a final centrifugation, the absorbances of the supernatants at 490 nm were measured using a spectrophotometer, and the astaxanthin contents were calculated as follows [26]:

astaxanthin (mg/L) *=* [4.5 × A_490_ × (V_a_ ÷ V_b_)]

where A_490_ is the absorbance at 490 nm of the supernatant, and V_a_ and V_b_ are the volumes of DMSO and microalga samples, respectively.

### 2.6. Statistical Analysis

Data are presented as means  ±  standard deviations. Statistical analyses were performed using one-way ANOVA followed by Tukey’s multiple comparison test using SPSS software (version 22; IBM Corp., Armonk, NY, USA). A value of *p* < 0.05 was taken to indicate statistical significance.

## 3. Results

### 3.1. Green Vegetative Stage of H. lacustris

**Figure 1:**
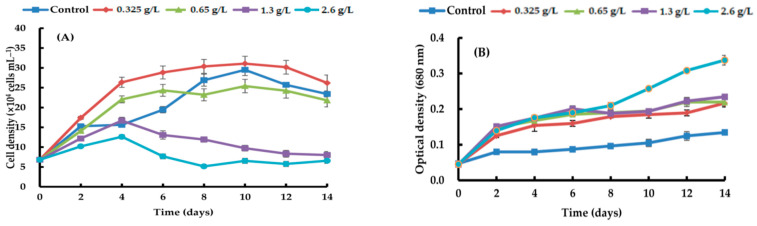
Comparison of growth curves of *H. lacustris* treated with different sodium acetate concentrations ((**A**): cell density, (**B**): optical density).

**Figure 2:**
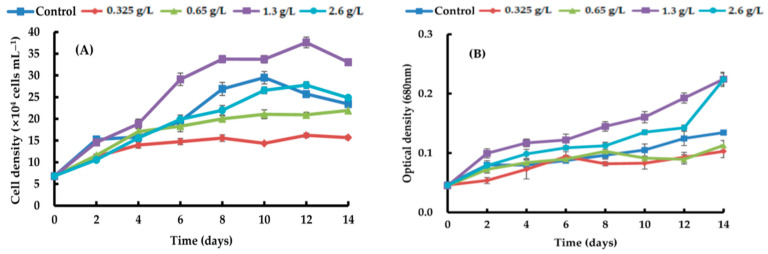
Comparison of growth curves of *H. lacustris* treated with different glycerol concentrations ((**A**): cell density, (**B**): optical density).

**Figure 3:**
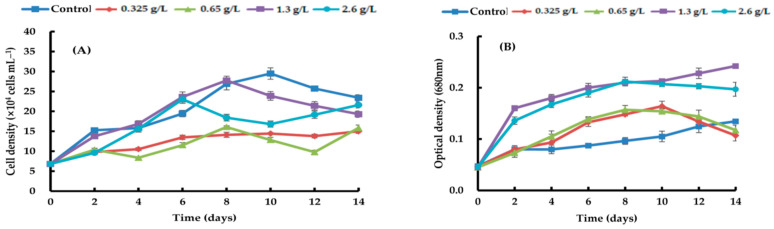
Comparison of growth curves of *H. lacustris* treated with different sodium gluconate concentrations ((**A**): cell density, (**B**): optical density).

**Figure 4:**
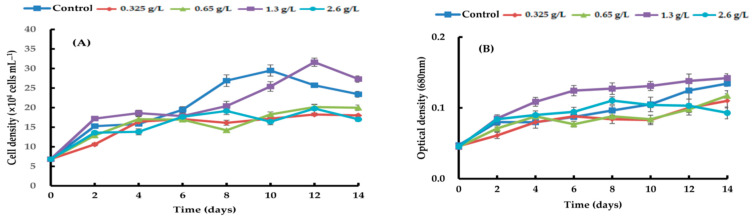
Comparison of growth curves of *H. lacustris* treated with different ribose concentrations ((**A**): cell density, (**B**): optical density).

By adding four different types of carbon sources—sodium acetate, glycerol, sodium gluconate, and ribose—at varying quantities, the growth efficacy of *H. lacustris* was confirmed. Generally, the growth curves of the cell numbers varied according to the concentration and source of carbon. At day 10, the maximum *H. lacustris* cell density of 31.08 ± 1.83 × 10^4^ mL^−1^ was achieved using 0.325 g/L sodium acetate (Figure 1). At 1.3 and 2.6 g/L sodium acetate, cells did not grow from day 4 and began to die. Glycerol-containing cultures grew at all concentrations. Specifically, after the 12th day of culture, the greatest increase in cell density was 37.61 ± 1.23 × 10^4^ cells mL^−1^ at a concentration of 1.3 g/L. Concentrations of 1.3 g/L and 2.6 g/L transitioned to the red stage in comparison to the control, and at the remaining concentrations, the growth curve’s amplitude was smaller than in the control (Figure 2). Regardless of the quantity, adding sodium gluconate led to lower cell densities than in the control (Figure 3), and by day 4, all of the cells were red. The addition of ribose continued to maintain the growth phase; partial or complete redness did not appear until day 12. As shown in Figure 4, ribose yielded a maximum cell density of 31.61 ± 1.06 × 10^4^ cells mL^−1^.

**Figure 5:**
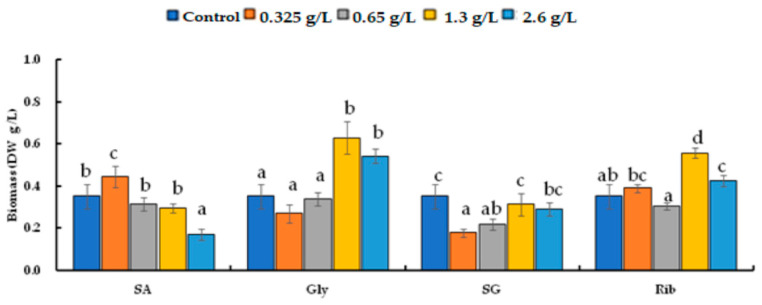
Comparison of DW after growing *H. lacustris* treated with different carbon sources and concentrations for 14-day culture (SA: sodium acetate, Gly: glycerol, SG: sodium gluconate, Rib: ribose). Mean values that do not share the same letter are significantly different at *p* < 0.05.

After growing for 14 days, the biomass was measured as DW. The cultures with glycerol and ribose had larger numbers of cells and higher DWs, and those with sodium acetate and sodium gluconate had lower DWs (Figure 5). The DW yields of the carbon sources differed significantly from the control (*p* < 0.05). The addition of 0.325 g/L of sodium acetate led to the highest DW of 0.446 ± 0.05 g/L. The addition of 1.30 g/L of glycerol resulted in a DW of 0.630 ± 0.08 g/L. Sodium gluconate reduced the DW compared to the control (0.352 ± 0.06 g/L). Ribose at 1.30 g/L resulted in the highest DW of 0.558 ± 0.03 g/L.

### 3.2. Astaxanthin Accumulation According to Growth Stage

**Figure 6:**
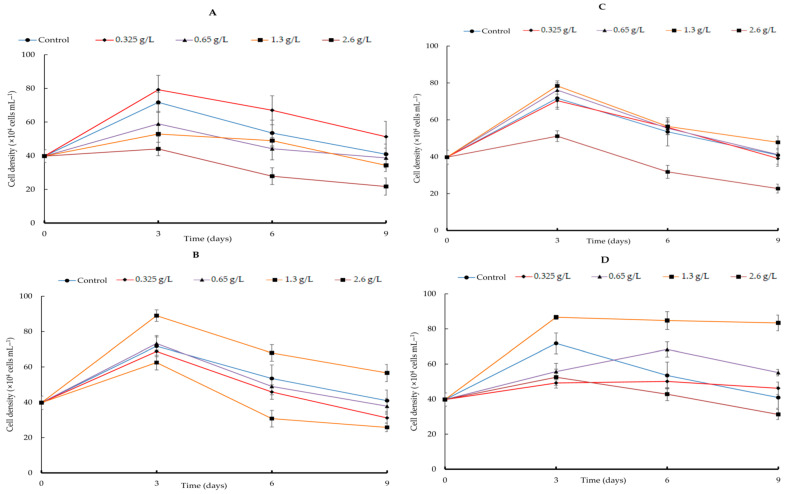
Comparison of growth curves of *H. lacustris* treated with different carbon sources and their concentrations in nitrogen-depleted cultures ((**A**) sodium acetate, (**B**) glycerol, (**C**) sodium gluconate, (**D**) ribose).

**Figure 7:**
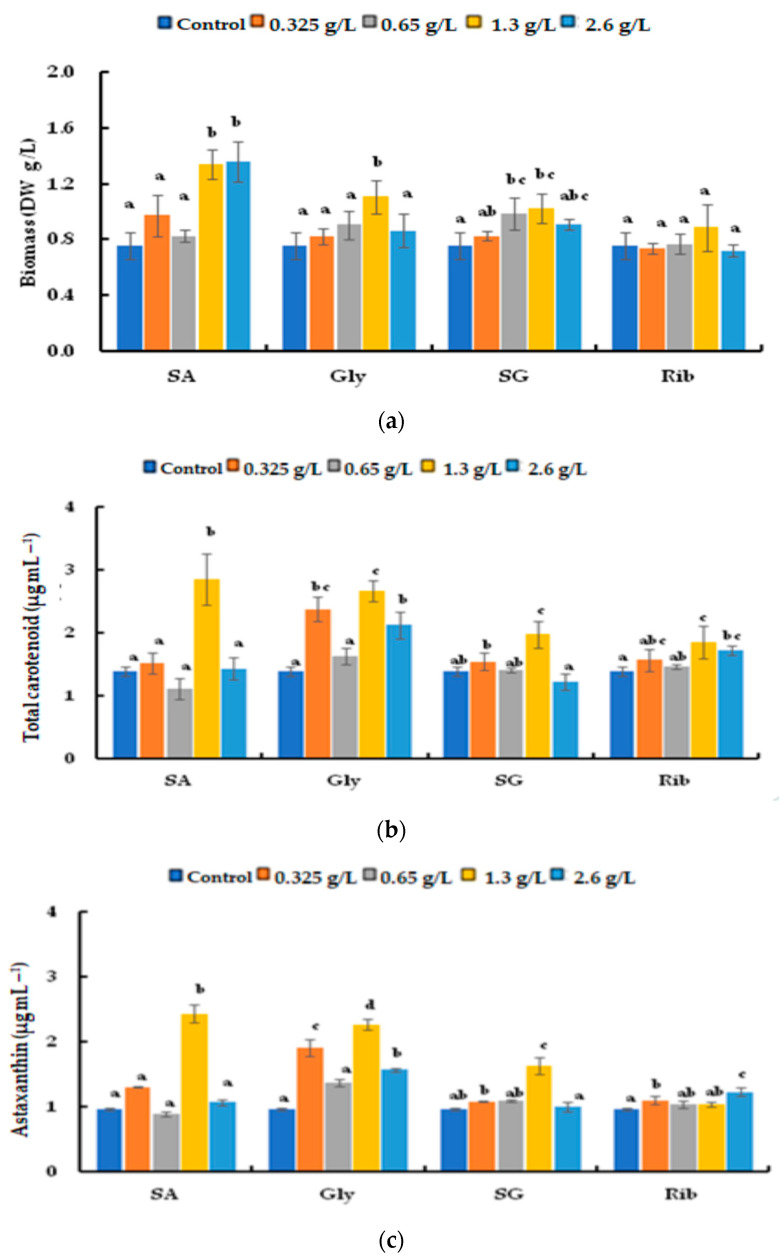
Comparison of (**a**) biomass, (**b**) total carotenoids, and (**c**) astaxanthin of *H. lacustris* treated with different carbon sources and concentrations under nitrogen-depleted cultures for 9 days (SA: sodium acetate, Gly: glycerol, SG: sodium gluconate, Rib: ribose). Mean values that do not share the same letter are significantly different at *p* < 0.05.

The rate of conversion to red non-motile astaxanthin-accumulating haematocysts was evaluated by adding an organic carbon source under nitrogen-depletion conditions (Figure 6). Figure 6 shows the growth of *H. lacustris* in the presence of the four carbon sources. The number of cells in all of the groups decreased after day 3. Beginning on day 3, the culture with sodium acetate gradually reddened; all cells were red by day 9. The culture with glycerol led to cells turning red on day 3; they remained in the palmella stage until day 9. The culture with sodium gluconate resulted in the reddening of cells on day 3. Similarly, cells cultured with ribose were green until day 6 and transitioned to the early palmella stage beginning on day 9. Figure 7 shows the biomass, total carotenoid content, and astaxanthin content on day 9. Biomass peaked after adding 1.3 and 2.6 g/L of sodium acetate at 1.34 ± 0.11 and 1.358 ± 0.14 g/L (DW), respectively. The highest biomass was achieved by adding 1.3 g/L glycerol and 0.65 g/L sodium gluconate, which did not differ according to ribose concentration.

The total carotenoid content was highest in cells cultured with sodium acetate (1.3 g/L), compared to a mean of 2.857 ± 0.40 µg mL^−1^ for the other carbon sources. Cells cultured with 1.3 g/L glycerol had the highest total carotenoid content of 2.672 ± 0.17 µg/mL, which was higher than in the control. The cells that were cultivated in the presence of sodium gluconate (except at 0.325 and 1.3 g/L) and ribose (except at 1.3 and 2.6 g/L) showed similar results as in the control. Cells cultured with 1.3 g/L sodium acetate exhibited the highest astaxanthin content (2.436 ± 0.14 µg/mL), and those cultured with glycerol had a higher mean astaxanthin content than in the control, with a peak of 2.269 ± 0.08 µg/mL at 1.3 g/L glycerol. Similarly, cells cultured with 1.3 g/L sodium gluconate had the highest astaxanthin concentration, 1.629 ± 0.13 µg/mL, but the other concentrations showed no difference from the control. Cells cultured with all ribose concentrations except 2.6 g/L had similar astaxanthin contents as in the control.

## 4. Discussion

Microalgal biomass can be a source of compounds for the nutraceutical, pharmaceutical, cosmetic, and biofuel industries [27,28]. Microalgae, particularly *H*. *lacustris*, are sources of natural products, including carotenoids, astaxanthin, lipids, carbohydrates, and proteins [29]. However, because of its slow growth rate and complex life cycle, large-scale cultivation of *H*. *lacustris* for commercial purposes is difficult [30]. Biomass yield can be improved by adding acetate and glucose to the *Haematococcus* culture medium [31]. There is a high chance of contamination with the addition of glucose in the culture of *H*. *lacustris* [32]. The introduction of exogenous carbon sources, such as sucrose in high-light conditions [33] and oxaloactate in combination with nitrogen stress [34], enhances astaxanthin accumulation in *H. lacustris*. While a lot of progress has been made in terms of improving astaxanthin accumulation, the majority of these developments are restricted to lab settings. Regretfully, there are still not as many *H. lacustris* mass cultivations at the industrial level [11]. The selection of an optimal organic carbon source is important because it is an important determinant of growth and astaxanthin accumulation [30,31]. Moreover, comparing the economic aspects of the costs of manufacturing and producing astaxanthin should be considered for industrial sustainability. An effective astaxanthin production strategy from *H*. *lacustris* requires an optimized cultivation strategy to boost astaxanthin accumulation and cell growth [35]. Astaxanthin accumulation in *H*. *lacustris* increased 2.7-fold under light stress and nitrogen deficiency during autotrophic growth [36].

In this study, four organic carbon sources increased the growth, biomass, and astaxanthin accumulation of *Haematococcus*. Among the different concentrations of sodium acetate, the highest growth was achieved with 0.325 g/L. Other concentrations resulted in reduced growth rates. In the nitrogen-depletion culture, astaxanthin accumulation was highest at a concentration of 1.3 g/L of sodium acetate. Cell growth and carotenoid content peaked with 0.75 g/L nitrate in the presence of 2% acetate [37]. Acetate enhances the growth and carotenoid production of *H*. *lacustris* [38]. However, the effect of acetate is concentration-dependent, with higher concentrations inhibiting growth but significantly increasing astaxanthin content per cell [39,40]. Excess acetate may enhance astaxanthin production by increasing the carbon/nitrogen ratio [41]. Sodium acetate affects homeostasis by causing an imbalance of calcium signaling and inducing the expression of carotenoid genes [42]. Supplementation of sodium acetate boosts metabolism (nitrogen, carbohydrates), synthesis of lipids, and energy usage [43]. Sodium acetate has been used as an organic carbon supplement during stress-induced secondary carotenogenesis at low pH [44].

Glycerol at 1.3 g/L increased the cell density by ≥1.5 fold of that in the control and resulted in the highest growth rate of the four carbon sources. The nitrogen-depletion cultures showed a similar trend to those with 1.3 g/L sodium acetate. The culture with 5 g/L glycerol increased the biomass by 34.22% [45]. Glycerol is reportedly superior to acetate in terms of enhancing *Haematococcus* biomass and astaxanthin production [46]. It is a polar and compatible solute for enzymes and membranes that readily enters algal cells by passive diffusion [47]. The addition of compounds with polyalcohol groups (such as glycerol, mannitol, sorbitol, and inositol) contributes to shortening of the lag phase, transition to the exponential phase, and prolongation of the growth period [35]. Glycerol enhances triacyl glycerol accumulation in *Haematococcus* and other microalgae. It increases the lipid content of *Nannochloropsis* and *Chlorella*, enabling their mixotrophic culture [48,49,50]. It is an inexpensive carbon and energy source for microalgae in mixotrophic regimes, and as a polar solute it readily enters algal cells by passive diffusion and is nontoxic to microalgae at high concentrations [51]. In a previous study, at 5 g/L, it increased the biomass productivity and triacylglycerol content of *Neochloris oleabundans*, *Botryococcus braunii*, and *Dunaliella* [52].

The addition of sodium gluconate, irrespective of concentration, resulted in a low growth rate, but the cells turned red after day 4. The amplitude of the growth curve of cultures with sodium gluconate, irrespective of concentration, was lower than that of the control, and the cells turned red after day 4. In nitrogen-depleted cultures, 1.3 g/L sodium gluconate maximized the astaxanthin content. Feeding gluconate during the mass cultivation of *H. lacustris* is a cost-effective way to improve cell activity and growth. Gluconate increases algal resistance to high light intensities, thereby promoting growth [32]. *H*. *lacustris* generally grows at low light intensities; at high light intensities, photosynthesis is inhibited, growth is stopped, and secondary carotenoids are produced [53,54]. The low growth in this study was a result of the need for a higher light intensity because of the presence of sodium gluconate. High light intensity favors the nutrient accumulation and growth of *H*. *lacustris* under gluconate mixotrophic conditions. Contradictorily, low light intensity coupled with gluconate addition could not trigger the growth of *H. lacustris* [32].

The addition of ribose at 1.3 g/L increased the growth rate compared to the control under nitrogen depletion conditions, but the cells did not change color irrespective of nitrogen depletion. Ribose promotes the production of NADPH and survival against nutrient deficiency and oxidative stress. C5 mannitols such as ribose are compatible solutes, organic osmotic agents, thermoprotectants, and antioxidants in algal cells [55,56]. The effects of ribose and acetate on photosynthesis in *H*. *lacustris* have been reported [23,24]. Ribose increased biomass content, cell count, and specific growth rate in *H. lacustris* [23]. The results from [23] indicate that the addition of ribose at a concentration of 1.15 g/L achieved 1.3-fold higher biomass than the addition of sodium acetate. However, to evaluate the implications of the addition of ribose from an economic standpoint, more research is still required [23,24]. Additionally, being too expensive is another drawback of using ribose [32].

In this study, we evaluated the effects of four organic carbon sources on astaxanthin and the accumulation of carotenoids. When attempting to improve growth and astaxanthin accumulation, separate *H*. *lacustris* cultures are needed because the growth conditions differ according to the organic carbon source used. However, the optimum light intensity varies according to the organic carbon source, so further research is needed. In this study, adding 1.3 g/L glycerol at the green stage raised biomass to the maximum level, whereas adding 1.3 g/L ribose raised biomass to the second highest level. Under nitrogen depletion conditions, the highest and second highest yields of astaxanthin were obtained from the cultures with the supplementation of sodium acetate (1.3 g/L) and glycerol (1.3 g/L), respectively. This study revealed that sodium acetate and glycerol produced the greatest improvements in biomass, total carotenoids, and astaxanthin accumulation under nitrogen-depletion conditions. Furthermore, we intend to use a unique open–closed hybrid model pond system to explore astaxanthin overproduction in mass cultures of *H. lacustris* and evaluate the effects of various organic carbon sources. We will use the findings to optimize the conditions for the industrial production of astaxanthin.

## Data Availability

Data are contained within the article.
